# Dynamic Causal Modeling of Preclinical Autosomal-Dominant Alzheimer’s Disease

**DOI:** 10.3233/JAD-170405

**Published:** 2018-09-11

**Authors:** Will Penny, Jorge Iglesias-Fuster, Yakeel T. Quiroz, Francisco Javier Lopera, Maria A. Bobes

**Affiliations:** a School of Psychology, University of East Anglia, Norwich, UK; b Department of Cognitive Neuroscience Cuban Neuroscience Center, Havana, Cuba; c Massachusetts General Hospital, Boston, MA, USA; d Group of Neurosciences, Medical School, Universidad de Antioquia, Medellin, Colombia; e Key Laboratory for Neuroinformation of Ministry of Education, Center for Information in Medicine, University of Electronic Science and Technology of China; f Wellcome Trust Centre for Neuroimaging, University College, London, UK

**Keywords:** Autosomal dominant, dynamic causal modeling, EEG, effective connectivity, multivariate

## Abstract

Dynamic causal modeling (DCM) is a framework for making inferences about changes in brain connectivity using neuroimaging data. We fitted DCMs to high-density EEG data from subjects performing a semantic picture matching task. The subjects are carriers of the *PSEN1* mutation, which leads to early onset Alzheimer’s disease, but at the time of EEG acquisition in 1999, these subjects were cognitively unimpaired. We asked 1) what is the optimal model architecture for explaining the event-related potentials in this population, 2) which connections are different between this Presymptomatic Carrier (PreC) group and a Non-Carrier (NonC) group performing the same task, and 3) which network connections are predictive of subsequent Mini-Mental State Exam (MMSE) trajectories. We found 1) a model with hierarchical rather than lateral connections between hemispheres to be optimal, 2) that a pathway from right inferotemporal cortex (IT) to left medial temporal lobe (MTL) was preferentially activated by incongruent items for subjects in the PreC group but not the NonC group, and 3) that increased effective connectivity among left MTL, right IT, and right MTL was predictive of subsequent MMSE scores.

## INTRODUCTION

Familial Alzheimer’s disease (FAD), due to dominantly inherited mutations in the presenilin 1 (*PSEN1*), *PSEN2*, and amyloid precursor protein (*APP*) genes, accounts for a small proportion (approximately 1%) of all cases of AD [1]. These mutations lead to AD in 100% of cases and the age of disease onset is similar between generations. This makes it possible to study the presymptomatic stage of the disease with a small number of subjects [2]. To achieve similar statistical power in an aging or at-risk population requires a much larger number of subjects.

The study of FAD has helped researchers identify the sequence of biomarker changes that precede symptom onset [2]. This has been facilitated, for example, by two large international projects; the Dominantly Inherited Alzheimer Network (DIAN) study [1], a US/UK/Australian project, and the Alzheimer’s Prevention Initiative (API), which studies a large Colombian kindred affected by the *PSEN1* E280A mutation [3].

Although M/EEG data is not routinely collected in the clinical management of ADs, there are nevertheless well-established effects at various stages of the disease [4, 5]. For example, in individuals with mild cognitive impairment (MCI), reductions in sensor-space N400 (see below) or P600 word repetition effects are associated with greater likelihood of subsequent transition to AD dementia [6]. Using EEG data from the Colombian kindred, Quiroz et al. [7] found differences in sensor-space event-related potentials (ERPs) between a Presymptomatic Carrier (PreC) group (carrying the *PSEN1* mutation) and a Non-Carrier (NonC) group. The PreC group showed less positivity in frontal regions and more positivity in occipital regions compared to NonCs. They hypothesized that control subjects may use frontally mediated processes to distinguish between studied and unstudied items whereas the PreC group uses visual details of the current item. Ochoa et al. [8] have found increases in effective connectivity, assessed using information theoretic measures, in the same PreC group during encoding of scene information.

This same Colombian kindred has been studied [9] with high-density EEG while performing a picture matching task, in which a first picture provided context and a second picture either matched or did not match that context. This paradigm elicited a characteristic change in the EEG signal—between matching and non-matching trials—400 ms after the presentation of the second picture, the so-called “N400”. EEG source reconstruction was then used to identify the anatomical locations of differences in the N400 between groups. They found smaller N400 s in right inferotemporal cortex and increased N400 s in left hippocampus and parahippocampus.

This paper uses dynamic causal modeling (DCM) [10] to identify the changes in brain connectivity that cause the ERP effects observed in the Bobes et al. [9] study. One of the motivations for revisiting this data is that it was acquired in 1999 and since then, follow-up cognitive assessments have periodically been made of subjects with the *PSEN1* mutation. This provides a fairly unique opportunity to find out whether changes in brain connectivity are predictive of future longitudinal changes in cognitive screening measures.

DCM is an established framework for making inferences about changes in brain connectivity using neuroimaging data and has been applied widely in cognitive and clinical neuroscience [11]. DCM for ERPs [12] uses a two-part forward model, the first part being a time series model describing how populations of neurons interact and the second-part being a spatial model describing how neuronal activity gives rise to EEG data. It then uses Bayesian methods to infer how neuronal pathways are differentially engaged as a function of experimental task or group.

The first goal of our DCM analysis is to identify the network architecture that provides a good explanation of ERP signals in the PreC group. Our second goal is to test whether the connections differ between PreC and NonC groups and our third goal is to see if any connections are predictive of subsequent Mini-Mental State Exam (MMSE) scores. There are many aspects to a DCM analysis and readers new to this area may benefit from the tutorial article by Stephan et al. [11].

### DCM and clinical applications

The goal of DCM is to make inferences about changes in effective connectivity, defined as the influence one neuronal system exerts over another. This influence may be mediated polysynaptically and so does not map one-to-one onto structural connectivity as measured, for example, by tract tracing or diffusion imaging [11]. Effective connectivity is a function of experimental task, with the configuration of network processing governed by activity in other regions (e.g., parietal/frontal) [13]. It is the changes in effective connectivity that are of primary interest in DCM.

DCM was first developed for fMRI data, for which it employed a simple “bilinear” model of neuronal dynamics and a well-established model of hemodynamics, and BOLD signal generation [10]. DCM was then developed for the analysis of ERP/ERF data and this extension will be described in detail in the following sections.

The majority of clinical applications of DCM have been made using fMRI and range from studies of aphasia [14], autism [15, 16], and major depression [17, 18] to Parkinson’s disease [19] and schizophrenia [20–22]. Further applications in psychiatry are reviewed in Yu et al. [23].

As the applications are too numerous to describe in detail, we focus on two. First, it has been shown that clinical groups can be differentiated using estimates of effective connectivity [14]. In this fMRI study of speech processing, connectivity estimates from a DCM of thalamo-temporal regions provided discrimination between moderately aphasic and healthy control groups that was better than that achievable using conventional activation-based and correlation-based methods. Second, DCM was used to study changes in brain connectivity due to an action selection task in two groups of subjects: control subjects and subjects with Parkinson’s disease who were undergoing dopaminergic therapy [19]. The optimal DCM was the same in both groups and showed modulation of coupling between prefrontal cortex and the pre-supplementary motor area (SMA). However, in a group of subjects with Parkinson’s disease who had withdrawn from medication, the optimal model revealed increased coupling between prefrontal cortex and a lateral premotor region. This finding corroborates independent evidence of a dopamine-dependent functional disconnection of the SMA in Parkinson’s disease.

We now turn to clinical studies using DCM for ERP/ERF based on EEG and MEG data, respectively. One high profile application using EEG is the study by Boly et al. [24] who found impaired top-down connectivity from frontal to temporal cortices in an auditory mismatch paradigm for subjects in a vegetative state. This impairment was not present in a group of control subjects or those in a minimally conscious state, thus demonstrating the importance of top-down signaling for conscious perception. Woodhead et al. [25] used MEG to study connectivity changes induced by a training program in a group of subjects with a stroke-induced reading deficit, and found increased connectivity among left hemisphere and reduced connectivity among right hemisphere regions. In later work [26], they used a similar approach in subjects with stroke-induced speech comprehension deficits and found that a phonological training program was superior to pharmacological intervention and acted by increasing connectivity between hemispheres.

Finally, we note that DCM for fMRI has been used to study differences in connectivity between control subjects and a group of subjects with MCI who went on to develop AD [27]. Subjects performed a visual attention task and analysis was restricted to regions in a cingulo-fronto-parietal network. They found that connectivity from a right middle frontal gyrus region was reduced in the MCI as compared to the control group and that this correlated with reductions of gray matter volume in that region.

## MATERIALS AND METHODS

This section begins by describing the subject groups, experimental task, EEG recordings, and follow-up cognitive assessments. We then describe the forward model in DCM for ERP which is based on a neural mass model of brain activity. The following sections then describe the Bayesian methods that DCM uses to fit this model to ERP data. We then describe our rationale for choosing the set of brain regions that enter the model and the statistical procedures for making inferences at the group level.

### Subjects

Participants were from a group of families with a history of FAD reported by Lopera et al. [28]. FAD in this population is caused with 100% penetrance by the E280A mutation in the *PSEN1* gene in chromosome 14. Participants were divided into two groups, the NonC group who were cognitively normal and did not carry the mutation, and the PreC group who carried the mutation but did not present cognitive dysfunction or dementia symptoms at the time of EEG acquisition (in 1999). This group can be more specifically described (at the time of EEG acquisition) as comprising cognitively unimpaired carriers [3]. The two groups were similar in sex and educational level and were matched on the Spanish version of the Barthel scale indicating that they functioned at a similar level in everyday activities. Subjects with a history of neurological or psychiatric illness were excluded from the study. See [9] for a complete description of study inclusion and exclusion criteria. Each participant gave their informed consent according to a protocol approved by the Human Subjects Committee of the University of Antioquia.

Individuals in the PreC group were expected to subsequently develop AD and so were monitored with neuropsychological tests in the intervening years (1999 to 2016). At the time of EEG acquisition, they were in a cognitively unimpaired preclinical stage.

Additionally, we only use subjects referred to in Bobes et al. [9] who had data recorded using a 120-electrode EEG system (see below). This was thought necessary to obtain accurate estimates of brain connectivity parameters. In the original study [9], there were 16 NonC and 17 PreC. Unfortunately, original epoched EEG data from 5 subjects were stored on DVDs which were corrupted since the original data acquisition. These subjects cannot be included in the current analysis. We therefore analyze data from 15 NonCs and 13 PreCs and are missing 1 NonC and 4 PreCs. There was no significant difference in the ages of our groups (*t* = 1.85, *p* = 0.07) with minimum, mean and maximum ages being 23, 42, and 50 for the NonC group and 25, 35, and 47 for the PreC group. The mean age of the subjects in the PreC group for whom the EEG data was no longer available, was 39. The MMSE scores for our PreC subject group at time of EEG scan (from most recent exam prior to that) had minimum, mean, and maximum values of 26, 28.4, and 30.

### Experimental task

Subjects viewed 118 pairs of drawings of objects and animals on a computer screen (see [29] for examples). Pairs of stimuli were selected in which 50% were semantically related (belonging to the same semantic category) whereas the other 50% were not. These are referred to as congruent (C) and incongruent (I) pairs, respectively. The drawings were sequentially presented, each for 1 s, the first pair member acting as context for the second.

The task of the subjects was to discriminate between the congruent and incongruent pairs of pictures by pressing one of two keys during the 2 s after the second stimulus offset. The experiment was designed to have a delayed response so that the EEG would not contain components of motor preparation.

### EEG recordings

EEG was digitally recorded at a sample rate of 200 Hz using a MEDICID-128 System (Neuronic, SA, Havana). Time series were bandpass filtered from 0.5 to 30 Hz (using a 5th order, two-pass, Butterworth filter) and a 60 Hz notch filter was used to remove mains signal. Data were epoched –100 to 900 ms around the presentation of the second picture in a pair. Epochs with generalized artefacts and eye-movements were removed as part of the initial study [9]. More specifically, before averaging, all EEG recordings were submitted to an automatic artifact detection procedure based on voltage threshold evaluation and EEG trials resulting from this procedure were visually inspected by a well-trained neurophysiologist, who checked the quality of the automatic detection and corrected it when needed. Channels with excessive noise were eliminated and substituted by interpolation from closest neighbors [9]. Congruent and Incongruent ERPs were then created by averaging over congruent and incongruent trials, but excluding trials that were classified incorrectly. ERPs were then baseline corrected by subtracting the average pre-stimulus amplitude and additionally low-pass filtered with a 18 Hz cut-off (zero phase distortion), consistent with [9]. The baseline correction used 100 ms of data to define the baseline but 900 ms to define the post-stimulus period. This asymmetry was also part of the initial study and is a standard processing procedure in ERP analysis [30].

### Cognitive screening

The cognitive functioning of subjects in the PreC group has been monitored since acquisition of the EEG data in 1999. The MMSE [31] has been administered at various time points between 1999 and 2015. The MMSE tests a number of different mental abilities including a person’s memory, orientation, attention, and language and is used extensively in dementia research to screen for cognitive impairment. Any score greater than 24 points (out of 30) indicates normal cognition. Below this, scores can indicate mild (19 to 23), moderate (10–18) or severe (less than 10) cognitive impairment. The MMSE is used primarily as a clinical measure for cognitive screening rather than a measure of cognition perse.

### Forward model

The forward model in DCM for ERP is based on a neural mass model of brain activity. This paper uses a modified Jansen-Rit model [32] to described neuronal circuit activity in each brain region, as proposed in the original DCM for ERP paper [12]. David et al. [12] describe how cortical units can be connected into hierarchical networks that follow known anatomical connectivity patterns [33]. This paper uses a 6-region model where *a*_*ij*_ denotes the strength of the connection from region *j* to region *i* and are stored in the matrix *A*. Details how these connectivity parameters affect network activity are provided (for a 2-region model) in Supplementary Material 1.

If we allow the connections between regions to vary with experimental condition (in this paper, congruency) then we can multiply each *a*_*ij*_ by a parameter *b*_*ij*_. Values of *b*_*ij*_ smaller/larger than unity reduce/increase the strength of the connection. These modulatory parameters are stored in a matrix *B*. We then collect all model parameters to be estimated in the vector *θ*. This includes the vectorized *A* and *B* matrices, and user-specified combinations of intrinsic connectivities and parameters of the firing rates and synaptic kernel functions (see Supplementary Material 1).

In Supplementary Material 1, we show how these convolution equations relate to differential equations (see also [12] for full details). The differential equations are then integrated for each experimental condition to produce time series of potentials for each population in each cortical unit, at *N*_*t*_ time points. In this paper, we model ERPs from zero to 500 ms post-stimulus onset. This is an atypically long time window for a DCM for ERP model, but such a long window is necessary to model the N400 (defined in [9] as lasting from 311 to 490 ms). The resulting ‘neuronal state matrix’ *X* (*θ*) is of dimension [*N*_*x*_ × *N*_*t*_]. The forward model is then specified as

(1) g(θ)=LX(θ)

Y=g(θ)+e

where *L* is an [*N*_*d*_ × *N*_*x*_] lead field matrix and *Y* are ERPs at *N*_*d*_ = 120 electrodes [12, 34] over the multiple experimental conditions. The lead field is defined using a concentric sphere model instantiated in the Statistical Parametric Mapping (SPM) software [34]. This forward model defines the likelihood *p* (*Y*|*θ*, *m*) where *m* specifies the model assumptions (e.g., which connections are modulated by congruency, as specified by the structure of the *B* matrix), *Y* is the ERP data, and *θ* are model parameters.

### Brain regions

The DCM for ERP framework explains the ERP signal as arising from a small number of brain regions. In this sense, it is more similar to equivalent current dipole (ECD) source reconstruction methods than ones producing distributed solutions over the whole of brain space (unlike both approaches it also has a model of temporal activity as described above). The selection of which brain regions to enter into a DCM can be made using either prior knowledge of the relevant brain regions from previous studies, or from univariate General Linear Model tests of functional specialization [11]. In this paper, we take a mixed approach.

Inferotemporal (IT) and medial temporal lobe (MTL) regions were chosen based on prior analyses by Bobes et al. [9]. This used a source reconstruction method based on a source space defined using a large number of dipoles, which were then partitioned into 68 anatomically defined areas. The optimal distribution of activity among combinations of these areas was then identified using Bayesian Model Averaging [35]. For the IT region, we use coordinates from Table 4 of Bobes et al. That is right (R) IT: [46,–54,–16] and we flip it for left (L) IT: [–46,–54,–16]. For the LMTL region, we take the coordinate of the left parahippocampus [–30,–22,–24] and flip it for RMTL [30,–22,–24]. These IT and MTL regions were selected as they were shown to exhibit congruency effects that differed between the NonC and PreC groups.

Additionally, DCM for ERP requires regions to receive input stimuli so that a signal can be evoked. As IT and MTL are high level processing regions and synaptically far away from sensory input, it was decided to include additional regions to receive input. The location of these regions was found using a group source reconstruction of activity in the 50 to 150 ms time window. This used the SPM implementation of group source reconstruction [36] that is in turn based on the multiple sparse priors (MSP) approach [37]. This is described in more detail in Supplementary Material 3 on Source Reconstruction.

We have used the term MTL to refer to brain regions whose coordinates were taken from the “parahippocampal” coordinates defined in Bobes et al. [9]. This is for two reasons. First, source locations are optimized during DCM estimation so will not remain in the same position (see Supplementary Material 3 on Source Reconstruction for what distance sources actually moved). Second, there is some controversy as to whether activity in deep brain regions can be recovered from EEG/MEG. But recent research, using Bayesian reconstructions with sparse priors rather than the distributed priors used earlier in the field, provide evidence that this is indeed possible [35, 38].

### Bayesian inference

**Fig.1:**
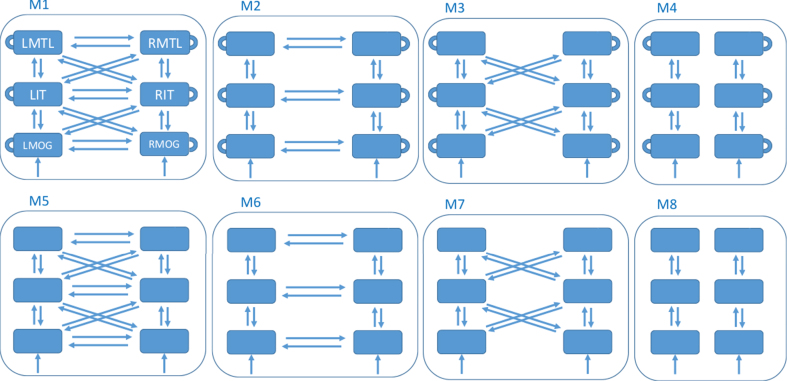
Model Space. All models have 6 nodes comprising the regions left and right medial temporal lobe (LMTL, RMTL), left and right inferotemporal cortex (LIT, RIT), and left and right middle occipital gyrus (LMOG, RMOG). The models differ as to whether they have within-region congruency effects (top versus bottom row - within-region effects are depicted as self-connections in the top row), hierarchical and lateral connections between hemispheres (column 1), lateral connections between hemispheres (column 2), hierarchical connections between hemispheres (column 3) or no connections between hemispheres (column 4). All models receive input, *u*, to bilateral MOG.

DCM then proceeds by defining a model space. This is a set of network structures, indexed by model *m* that define which brain regions are connected and which (within and/or between region) connectivity parameters are modulated by experimental factors. Our factor is congruence. This paper defines a model space with 8 different models, as described in Fig. 1. This model space was designed after preliminary analyses suggested that data in our rather long (for DCM for ERP) time window might be better modeled with hierarchical connections between hemispheres, rather than the purely lateral connections used in previous studies. We set up the model space to formally test this hypothesis.

For each model, we have a prior distribution over parameters, *p* (*θ*|*m*), which for example constrains parameters to lie within a physiologically plausible range. Additionally, DCM for ERP allows for a prior distribution over source locations [39] which allows the final (posterior) locations to vary over subjects. Here we used the default values of 4 mm for the prior variance of each *x*, *y*, *z* source location parameter. The priors over network parameters are set to the default values described in [12].

Given ERP data *Y*, DCM then uses Bayesian inference to compute a posterior distribution over model parameters

(2) $$p(\theta |Y,m)=\frac{p(Y|\theta )p(\theta |m)}{p(Y|m)}$$

where the denominator is referred to as the model evidence given by

(3) p(Y|m)=∫p(Y|θ,m)p(θ|m)dθ

In DCM the likelihood, *p* (*Y*|*θ*, *m*), is defined by integrating the differential equations in Supplementary Material 1 (on Neural Mass Models) to produce a prediction *g* (*θ*), such that better fits between predictions and empirical data have higher likelihood. For nonlinear models such as DCM, the posterior distribution over parameters cannot be computed analytically (using Equation 2) but must be approximated. DCM uses the Variational Laplace (VL) algorithm [40] to do this which provides an estimate of the posterior mean connection values {*A*^*MP*^, *B*^*MP*^} and the log model evidence, log *p* (*Y*|*m*). A second goal of Bayesian inference is to compute the posterior density over models

(4) $$p(m|Y)=\frac{p(Y|m)p(m)}{p(Y)}$$

Given any two models to compare (and uniform priors) we can use Bayes factors

(5) $$B_{\mathrm{ij}}=\frac{p(Y|m=i)}{p(Y|m=j)}$$

One can then derive that

(6) $$p(m=i|Y)=\frac{1}{1+\exp(-\log B_{\mathrm{ij}})}$$

leading to the relationship that a log Bayes factor of 3 corresponds to a posterior model probability of 0.95 (in favor of model *i* over *j*). Just as a culture has developed around the use of *p*-values in classical statistics (e.g., *p* < 0.05), so one has developed around the use of Bayes factors. Raftery [41], for example, notes that log Bayes factors greater than 3 (or 5) provide strong (or very strong) evidence in favor of model *i*.

### Group and family inferences

Having computed the posterior mean connection values, $\theta_{\mathrm{MP}}^{i}$, and log model evidences, log *p* (*Y*^*i*^|*m*), for each subject and model one can then make inferences at the group level.

For inferences about models, we use Fixed Effects Bayesian Model Comparison at the group level [42] which assumes that the optimal model is the same for all subjects in a group. Here, one uses the Group Bayes Factor which is simply the product of Bayes Factors over subjects in the group (so the log Group Bayes Factor is the sum of the logs of the Bayes factors over subjects).

If one has several models in the hypothesis space, then it can be useful to aggregate models into families [43]. We decompose the model space used in this paper (shown in Fig. 1) in two ways. First, we place the top and bottom row models into separate families, and family level inference [43] here allows one to test the hypothesis that it is useful to allow intrinsic connectivity to be different for congruent versus incongruent trials. Second, the four columns in Fig. 1 which make different assumptions about inter-hemispheric effective connectivity are placed into four families. Family inference here then allows one to infer which is optimal for our data. These family inferences are analogous to testing for main effects of factors in an analysis of variance.

For inferences about parameters, there are two approaches. The Summary Statistic approach [44] is implemented by applying classical inference on the relevant components of *A*^*MP*^ or *B*^*MP*^ over all subjects in the group (using regression or one-sample *t*-tests) or between groups/effects (using two-sample *t*-tests). A drawback of the Summary Statistics approach is that it does not take into account the uncertainty in the estimated parameters for each subject (or, indeed, the correlations among them). An alternative recently developed approach, Parametric Empirical Bayes (PEB) [45], does accommodate this uncertainty, and we apply it to our group level inferences. This PEB framework has been used with DCM, for example, to explain between-subject variability in visual gamma activity using MEG [46].

Specifically, we first use PEB to test for group differences in connectivity parameters and then use that subset of parameters to predict subsequent MMSE scores. This latter prediction uses a Leave-One-Out (LOO) cross validation procedure, a standard approach in statistics [47], in which a model is fitted to data from all but one subject and a prediction of the score is made for that subject. In our case, this is a multivariate linear model as described in previously published work [45]. This operates in turn for all subjects and reports Pearson’s correlation and the corresponding classical *p*-value between predicted and empirical scores.

### EEG data summary

This study uses data from 28 subjects, 13 of whom are carriers of the *PSEN1* mutation (the PreC group) and 15 of whom are not (the NonC group).

For each subject, 118 pairs of images were presented (59 congruent and 59 incongruent). The study uses EEG data epoched around the presentation of the second image in each pair. The EEG epochs were then averaged over (correct trials only) to produce an ERP for each condition (congruent and incongruent). EEG signals were recorded from 120 channels. We restrict our DCM analysis to the time window leading up to and including the N400 that was the subject of previous analysis by Bobes et al. [9]. DCM analysis therefore used 100 time points for each channel, between 0 and 500 ms relative to the presentation of the second image (signals after 500 ms were not modeled as they do not contain information characteristic or predictive of the N400).

For each subject, we therefore have 2 ERPs (congruent and incongruent), each of which has 120 spatial dimensions (in sensor space), and 100 temporal dimensions (over the peristimulus time period 0 to 500 ms). These are the ERP data to which the DCMs are fitted.

## RESULTS

### Behavioral data

A ’hit’ is a correct recognition of a congruent item, and a ‘false alarm’ an incorrect recognition of an incongruent item. Bobes et al. [9] report the following rates: 76% hits and 9% false alarms for NonC, and 72% hits and 6% false alarms for PreC. The difference between PreC and NonC was not significant. For the subset of subjects studied in this paper the corresponding figures are; 89% hits and 14% false alarms for NonC (sensitivity index [48], *d*’ = 2.44) and 90% hits and 15% false alarms for PreC (*d*’ = 2.54). Again, there is no significant difference between the two groups (two sample *t*-test based on *d*’ scores, *p* = 0.26, *t* = 1.14).

In the PreC group, age and task performance (computed using *d*’) were negatively correlated (*r* = –0.69, *p* = 0.01) meaning that younger subjects performed better. There was no such correlation in the NonC group (*r* = 0.26, *p* = 0.37) or collapsed over both groups (*r* = –0.27, *p* = 0.17).

### Cognitive screening

**Fig.2:**
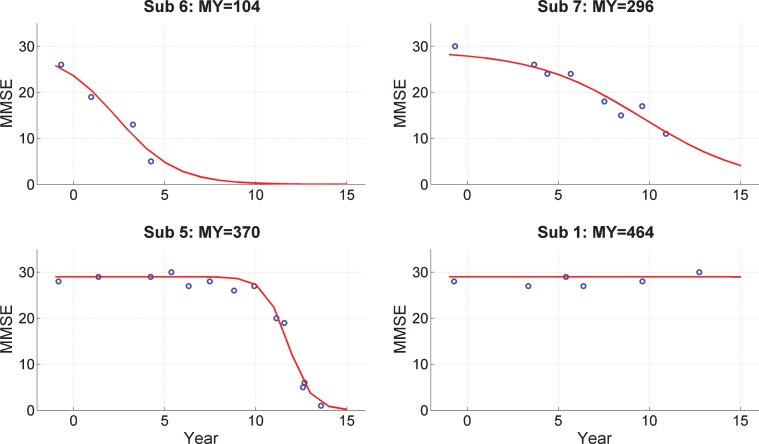
Trajectories of Mini-Mental State Exam (MMSE) scores. MMSE trajectories during follow-up period for 4 subjects from the PreC group. The x-axis labels Year with 0 corresponding to 2000. The EEG data were acquired in 1999. Blue dots denote empirical MMSE scores and the red line indicates the trajectory estimated using a logistic decay model. The MY values above each plot correspond to MMSE-Years, computed as the integral under the curve. References to color relate to the online version of this article.

Figure 2 shows MMSE “trajectories” in the follow-up period for 4 subjects selected to show a representative variety of changes (e.g., sudden, gradual, or no change). Trajectories for the remaining subjects are provided in Supplementary Material 2 (on Cognitive Trajectories). We fitted a Logistic Decay model to each subject’s scores having the mathematical form

(7) $$y=m_{0}\left[1-\frac{1}{1+\exp(-b(t-a))}\right]$$

where *y* is MMSE score, *t* is time, *a* and *b* are parameters to be estimated, and *m*_0_ is the MMSE score at the time of EEG acquisition which is taken to be 28. Model fitting was implemented using the same VL algorithm used to fit the DCMs. The model fits are shown as the red curves in Fig. 2.

From these model fits we then compute the quantity MMSE-Years, *MY*, which is the integral under the fitted curve. Given the 16-year interval (from 1999 to 2015) and a maximal potential MMSE score of 30, the maximum value for MY is 480. This is nearly obtained for subject 1 and four others (see Supplementary Material 2) who do not show a decrease in MMSE values during follow-up.

The MY scores were then normalized to have zero mean and unit variance across the group. These normalized values are then regressed onto DCM parameter estimates as described below. MMSE-Years and age were negatively correlated (*r* = –0.73, *p* = 0.005) meaning that older subjects had lower values (in the 16-year time horizon post EEG collection). MMSE-Years and task performance (as measured using *d*’) were positively correlated (*r* = 0.72, *p* = 0.006) meaning that subjects who were better at the task had higher MMSE-Years values.

### Source reconstruction

Group source reconstruction of activity in the 50 to 150 ms time window (see Supplementary Material 3 for details) led to the identification of the brain regions left middle occipital gyrus (LMOG): [–28,–86,30] and RMOG: 28,–86,30. Here we have taken the LMOG coordinates from the peak of the statistical parametric map of the group source reconstruction. The RMOG coordinates in the map were not exactly homologous (i.e., same *y* and *z* coordinates), but close, so for consistency with the other region definitions, here we set the RMOG coordinates to be symmetric to LMOG. Together with LIT and RIT and LMTL and RMTL, we therefore have six brain regions in our DCMs.

### Model comparison

We now address the issue of how brain regions are connected and how that connectivity varies with congruency in the PreC group. We consider eight different types of architecture (see Fig. 1) and allow all connections within each to change with congruency. In DCM terminology [10], the *A* matrix contains connectivities associated with the congruent condition and the *B* matrix contains changes in connectivity due to incongruent versus congruent conditions. Thus, for a connection with no effect of congruency, the *B* value is unity and the value of the connection for both conditions is the value in the *A* matrix.

We first test for the effect of between hemisphere connectivity (columns of Fig. 1) using family level inference [43], i.e., collapsing across within-region congruency (see Group and Family Inferences for a description of model families). This revealed that hierarchical connections between hemispheres are best, followed by hierarchical and lateral (log GBF = 1935), followed by no hemispheric connectivity (log GBF = 6460). As these log Bayes factors are larger than 5 we can conclude these effects are very strong (see Bayesian Inference section).

We then compared the two models in the winning family. Bayesian model comparison revealed the best model to be M7 which assumes hierarchical connections between hemispheres and no within-region congruency effects. Log Group Bayes Factors (GBF) in favor of M7 over M3 are 31.7.

Additionally, we compared all models without collapsing across columns. This showed the best model to be M7 followed by M3, which in turn is followed by the third best model M5, with a log GBF of 794 in favor of M3 over M5. All of these models (M3, M7, M5) have hierarchical connections between hemispheres.

For completeness, we also report comparisons for the NonC group. Testing for the effect of between hemisphere connectivity revealed that hierarchical connections between hemispheres are best, followed by hierarchical and lateral (log GBF = 65), followed by no hemispheric connectivity (log GBF = 840). This ordering is the same as for the PreC group. Comparing the two models in the winning family, however, revealed M3 to be the best rather than M7 (log GBF = 670). This is not the same as PreC. Finally, the comparison across models without collapsing across columns revealed the best model to be M1, followed by M3, M8, and M7. The results in the following sections are based on the optimal model for the PreC group, M7. Before proceeding to these sections, we first summarize a few characteristics of these models. Firstly, there were no between group (PreC versus NonC) or condition (congruent versus incongruent) differences in the accuracy with which the models fitted the data. Second, there were no between group differences in the distances which the sources moved during model fitting. Interestingly, however, the MOG sources moved significantly less than the IT and MTL sources, perhaps as a consequence of their prior location being based on the current data set and source space definition (whereas prior locations from IT and MTL were taken from [9]). More detailed reports of model fits are provided in Supplementary Material 4 (on DCM Diagnostics).

### Effects of group and congruency

This section reports effects of group and congruency on parameter estimates as revealed using PEB. We first ran PEB using the A matrix values from NonC and PreC groups. Connections that were significantly different between groups are reported in Table 1. The connections showing the strongest group effect are RIT to RMTL and LMTL to RIT. Both are larger in the PreC group.

**Table 1:** A matrix connections showing effect of group

Pathway		Group Means, ā		Statistics
From	To	NonC	PreC	*P*_*post*_
LMOG	LIT	0.81	0.99	0.98
RMOG	LIT	1.10	0.86	0.99
RIT	RMTL	0.90	1.18	1.00
LMTL	RIT	0.82	1.12	1.00

Connections in this table have a posterior probability, *P*_*post*_, greater than 0.95 of showing a group difference.

**Fig.3:**
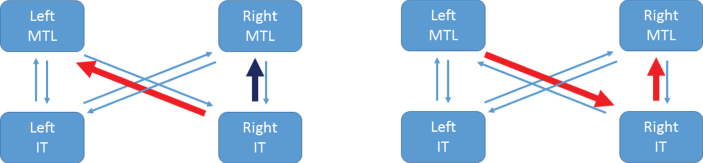
Congruency and correlation effects in the PreC group. The left panel illustrates that the RIT to LMTL pathway is strengthened for incongruent items (red arrow) whereas the RIT to RMTL pathway is weakened for incongruent items (dark blue arrow). The right panel illustrates the two connections that are significantly larger in the PreC than NonC groups and show a significant correlation with MMSE-Years (red arrows). References to color relate to the online version of this article.

Table 2 shows equivalent results for B matrix values. The entries here indicate that the RIT to LMTL pathway is strengthened for incongruent items in the PreC group, but weakened in the NonC group. Whereas, the RIT to RMTL pathway is weakened for incongruent items in the PreC group but hardly different in the NonC group. This is depicted for the PreC group in Fig. 3 (left panel).

**Table 2:** B matrix connections showing effect of group

Pathway		Group Means, *b*		Statistics
From	To	NonC	PreC	*P*_*post*_
RIT	LMTL	0.81	1.06	0.99
RIT	RMTL	1.01	0.78	0.99

Connections in this table have a posterior probability, *P*_*post*_, greater than 0.95 of showing a group effect on the modulatory parameter. That is, a group by congruency interaction.

### Predicting subsequent cognitive screening measures

We then used the four A matrix connections in Table 1 to predict MMSE-Years using a multivariate linear model. The accuracy of this model was assessed using LOO cross-validation. This showed a significant correlation (r^2^ = 0.23, *p* = 0.048) meaning that estimates of effective connectivity can predict MMSE-Years.

We then used the two B matrix connections to predict *MY* using the same procedure, but this produced a null result (r^2^ = 0.02, *p* = 0.68). This shows that the differential engagement of these pathways (for congruent versus incongruent items) is not predictive of MMSE-Years.

Examining our positive results (with the A matrix entries) in more detail we then applied the LOO procedure to a single connection at a time. These univariate correlations and the significance thereof are shown in Table 3. Only RIT to RMTL and LMTL to RIT are significant univariate predictors of *MY*. Using the two connections that showed the strongest group effect (RIT to RMTL and LMTL to RIT) together gives r^2^ = 0.44, *p* = 0.007. These two pathways are highlighted in Fig. 3 (right panel). For completeness, we also present tests of group, congruency and correlation with *MY* using the summary statistic approach in Supplementary Material 5 (on Parameter Inferences).

**Table 3:** Correlations with MMSE-Years for A matrix connections showing group effect

Pathway		Statistics	
From	To	*R*^2^	*p*-values
LMOG	LIT	0.005	0.59
RMOG	LIT	0.14	0.89
RIT	RMTL	0.28	0.03
LMTL	RIT	0.41	0.009

*R*^2^ and *p*-values computed from leave-one-out cross validation. Only RIT to RMTL and LMTL to RIT are significant univariate predictors of MMSE-Years.

**Fig.4:**
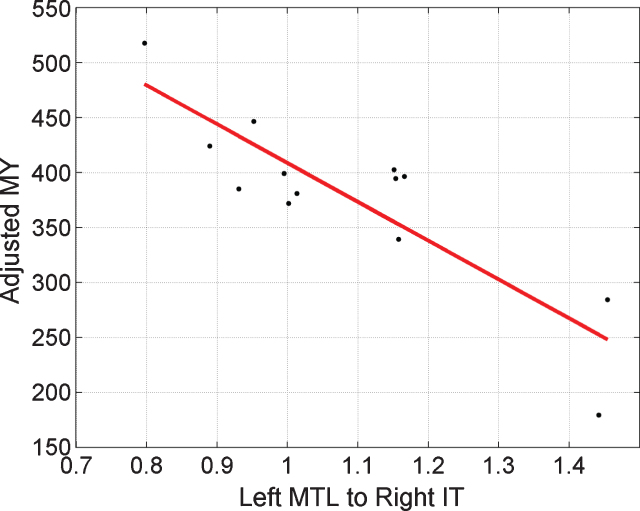
Regressing MMSE-Years onto brain connectivity. Stronger activation of the Left MTL to Right IT pathway, ā, is associated with smaller *MY* values. A value of ā = 1 corresponds to the prior mean value. Here, the x-axis corresponds to pathway strength for congruent items. Adjusted MY is the MY score computed as the area under the MMSE trajectory curves (over the period 1999 to 2015) shown in Fig. 2 but adjusted for the effects of age and performance using multiple regression as described in Supplementary Material 5. The Left MTL to Right IT value is a parameter of a DCM fitted to EEG data acquired in 1999.

Given the correlations between MMSE-Years and age/performance reported above, we performed additional analyses controlling for these effects. This is especially important as a recent study has reported an association between AD biomarkers and age in the Colombian cohort [49]. Specifically, we set up a PEB model with group level regressors being connection value, age, and performance. The same LOO cross-validation procedure was then used to assess significance of the (partial) correlation between connection value and MMSE-Years. Using all four of the connections that show a group effect, we have *r* = 0.61, *p* = 0.01. For the LMTL to RIT connection alone, we have *r* = 0.85, *p* = 0.0001, but for RIT to RMTL alone, we have *r* = 0.09, *p* = 0.38, this latter effect being no longer significant. We also implemented multiple regressions using summary statistics (as described in Supplementary Material 5) which showed that both correlations remained significant. Figure 4 plots MMSE-Years adjusted for the effects of age and performance, versus the strength of the LMTL to RIT pathway.

Figure 3 summarizes the main results of this section and the previous section. Other factors being equal, the increased RIT to LMTL connection for incongruent items in the PreC group would cause hyperactivation of the LMTL region.

## DISCUSSION

### Hierarchical connections between hemispheres

Our first finding was that the optimal neuronal architectures for both the presymptomatic and control groups had hierarchical connections between hemispheres. This was revealed by family-level comparisons over model space in which families either had lateral connections between hemispheres, hierarchical connections between hemispheres, both, or no hemispheric connections.

In the DCM literature to date, only models with purely lateral connections between hemispheres have been used (but see [50] for an exception with the same connectivity as M7). These purely laterally connected models were not optimal for our data. We hypothesize this is because we are modeling ERPs with very late components. Our models have a peristimulus time going out to 500 ms which exceeds that of any previous DCM for ERP application (to our knowledge the longest peristimulus time period of a previously published DCM for ERP/ERF study is 400 ms [24, 50]). Thus it may be that hierarchical connections between hemispheres (as part of a strongly recurrent network) are necessary for explaining very late components in ERPs. It could be the case, however, that very late components are equally well modeled by deeper networks (e.g., with 4 rather than 3 hierarchical levels in each hemisphere, perhaps including a frontal region) or by employing different local neuronal circuit models in each brain region. For a review of the local neuronal circuit options now available in DCM for ERP, see Moran et al. [51].

Anatomical evidence for hierarchical connections between hemispheres is provided from tract tracing studies in non-human primates [52] and, for a subset of brain regions (e.g., hippocampus/parahippocampus) as revealed by diffusion imaging [53]. But there are no major direct anatomical pathways between RIT and LMTL. This is not a concern, however, as between-region connections in DCM are effective connections, where effective connectivity is defined as the influence one neural system exerts over another and can be mediated polysynaptically [11]. This allows cognitive neuroscientists to construct models with a small number of regions that do not have all intermediate connecting levels. The alternative would be to create very large network models with most regions merely acting as relay stations, and not changing their activity as a function of experimental condition. A reasonable objection here is then why concern oneself with structural connectivity within each region but not in the network as a whole. An approach with detailed structural connectivity at both levels is provided by the Virtual Brain Project [54], but currently no methods exist for fitting these models to empirical ERP data.

### Group by congruency interaction

We found two pathways showing a group-by-congruency interaction. First, RIT to RMTL is reduced for incongruent items in PreC but shows little change in the NonC group. This implies that, other factors being constant, RMTL does not show a hyperactivation effect in our PreC group.

Second, RIT to LMTL is increased for incongruent items in the PreC group, but reduced in the NonC group. This is consistent with other findings in the literature of left hippocampal hyperactivation in preclinical or MCI-stage AD, which we now briefly review.

Mondadori et al. [55] found increased fMRI activation in left frontal, temporal, and parietal neocortices and in left hippocampus during the learning and retrieval of an episodic memory task (pairing of unknown faces with professions). Sperling et al. [56] reviews studies, some of which show increased activation of the hippocampus and related structures within MTL with respect to controls, when encoding new memories.

Additionally, Quiroz et al. [57] have found hyperactivation of the hippocampus during encoding. They used a face-name associative memory task in presymptomatic individuals with the *PSEN1* mutation (drawn from the same Colombian cohort as in the Bobes et al. study). Functional MRI results showed greater activation of the right anterior hippocampus during presentation of novel face-name pairs in the presymptomatic group as compared to a control group. No group differences were found for familiar face-name pairs (i.e., recognition).

Bookheimer et al. [58] used a memory task, involving unrelated word pairs, in two healthy populations of subjects. One population carried the A4-allele of the APOE gene, placing them at high-risk of later developing AD, and the other population carried the healthy allele. Functional MRI results showed greater bilateral hippocampal activation in the at-risk group during memory recall. Greater hippocampal activation correlated with worse memory performance at 2-years follow up. Additionally, widespread increases in activation were found in the at-risk versus control group (A3-allelle) in multiple left hemisphere brain regions. See Rao et al. [59] for a recent longitudinal study that elaborates on these findings (APOE4 carriers and non-carriers scanned at 3 time points).

### Consistency with previous analyses

This paper is based on previous work by Bobes et al. [9]. A main focus of their analysis is the N400 which reflects increased neuronal activity for incongruent versus congruent items in a time window around 400 ms post-stimulus. They found smaller N400 s in RIT and increased N400 s in left hippocampus and parahippocampus. Given that we used the same anatomical coordinates for these sources (our RIT is the same as their RIT, our LMTL is the same as their left parahippocampus) one would hope to recover the same effects.

The group-by-congruency interaction we found indicates that the RIT to LMTL pathway is strengthened for incongruent items in the PreC group, whereas it is weakened in the NonC group. Other factors being equal, this will lead to greater activation of LMTL for incongruent items in PreC subjects. This is therefore consistent with Bobes et al. [9], has also been found in other studies (see above).

However, we found no evidence for the reduction of the N400 in RIT. This is a concern, but there are two reasons why this may have occurred. First, we are not using exactly the same set of subjects, as some data stored on DVD had been corrupted since the initial study (17 years ago). Second, although DCM operates in source space the definition of this space is different to that in [9]. Our sources live on one of SPM’s canonical surface meshes (the one with 8192 dipoles) and Bobes et al. used 20,092 dipoles constrained to the gray matter of the Montreal Neurological Institute (MNI) brain (neither approaches used subject-specific MRIs to define source space). Thus we may be reporting on the activations of different populations of cells (or their ECD).

It is possible that had we chosen different prior locations for our six brain regions that different results would have been obtained. The locations of the MOG regions were based on statistical tests in SPM source space. But the locations of the MTL and IT regions were taken from the Bobes et al. study. With hindsight, perhaps it may have been better to also choose MTL and IT locations from statistical tests in SPM-source space (e.g., tests of group and congruency in selected time windows). However, this may not have made a difference. In DCM for ERP, the locations are optimized during the model estimation process (within bounds specified by the prior) and we found that the sources in the four MTL/IT regions moved significantly more than the MOG regions (see Supplementary Material 4), perhaps reflecting that their locations had not been set so well *a priori*, but correcting for this during model fitting. An alternative strategy here would be to define priors over source locations based on a preliminary stage of ECD modeling, as used in a previous DCM for ERP study [60].

The DCM for ERP framework allows imaging neuroscientists to propose multiple models of ERP data and these models can differ in the number and location of brain regions. The Bayesian model evidence can then be computed for each and provides an objective measure, balancing model fit and complexity, for adjudicating as to which is the optimal solution [12]. Future studies could fit DCMs for ERP to the same data to see if, e.g., deeper networks have higher model evidence.

### Group and MMSE-Years

Collapsing across congruency, we found four pathways that were differentially activated in the PreC versus NonC groups. These are i) LMOG to LIT, ii) RMOG to LIT, iii) LMTL to RIT, and iv) RIT to RMTL. We then used these four estimated connection values in each subject to build a multivariate predictor of MMSE-Years and found a significant correlation between predicted and empirical values. Looking at this in more detail, we then found that only pathways (iii) and (iv) made a significant contribution to this prediction. Controlling for age and performance, only pathway (iii) showed a significant correlation. We therefore infer that the strength of the LMTL to RIT pathway is associated with the disease process.

Pathways (iii) and (iv) are both larger in the PreC than NonC groups and the larger they are in the PreC group the smaller the MMSE-Years value. This is somewhat similar to the results of Miller et al. [61] who found that greater left hippocampal activation for novel versus repeated scenes predicted subsequent cognitive decline.

### Closing thoughts

We now turn to more speculative thoughts based on our findings. The stronger connections referred to above could be a consequence of functional compensation [62]. If this is the case, then these large connections may be necessary for presymptomatic subjects to perform the tasks to the same level as control subjects, despite underlying neurodegeneration. In this sense, these strong connections are a good thing.

Alternatively, the larger connections may be a result of runaway synaptic plasticity [63]. These large values may then themselves be the cause of subsequent neuronal damage via a number of possible mechanisms. For example, the resulting increased excitatory drive could contribute to cell death in these regions (e.g., LMTL) via excitotoxicity. Additionally, there is evidence that increased neural activation is correlated with increased amyloid deposition, a response which may be protective in the short term (as it is thought to reduce plasticity) but damaging in the long term [63]. If this view is correct, one might then hope to use longitudinal EEG and DCM for ERP as part of a drug intervention program in which the timing and dosage of, for example, memantine [64] (which acts to reduce plasticity [65] and functional connectivity [66]) could be used to reduce these connection strengths, and so slow disease progress.

The results in this paper suggest a model in which altered effective brain connectivity relates to cognitive processes in a population of FAD patients. However, further studies must be made before applying the model to future clinical studies of cognitive decline in FAD. For example, it is unclear how robust the findings are with respect to source estimation parameters, specification of post-stimulus ERP period, and network features (e.g., number and localization of nodes in the model). Future studies systematically manipulating these parameters are therefore needed. Additionally, network analyses based on fMRI recordings from the same population of subjects would provide an essential cross-validation.

## Supplementary Material

Supplementary Material 1

Supplementary Material 2

Supplementary Material 3

Supplementary Material 4

Supplementary Material 5
