# Supplementary material for: Dynamic Causal Modeling of Preclinical Autosomal-Dominant Alzheimer’s Disease
Source: J Alzheimers Dis. 2018 Sep 11;65(3):697–711. doi: 10.3233/JAD-170405 (PMC6923812; doi:10.3233/JAD-170405)
Supplement: Supplementary Material 1 [file jad-65-jad170405-s001.pdf]

Supplementary Material 1
Dynamic Causal Modeling of Preclinical Autosomal-Dominant Alzheimer's Disease

Neural Mass Models

Neural Mass Models (NMMs) of a single cortical region have been proposed by Jansen and Rit [1] and a modified version incorporated into DCM for ERP [2]. Postsynaptic potentials (PSPs) at excitatory synapses are related to firing rates via convolutions with synaptic kernels

$$v_{out}(t) = h_e(t) \otimes s(v_{in}) \tag{1}$$

where $\otimes$ is the convolution operator and the population firing rate function

$$s(x) = \frac{1}{1+\exp(-r_1(x-r_2))} - \frac{1}{1+\exp(r_1 r_2)} \tag{2}$$

has parameters r_1 and r_2, and the synaptic kernel is given by an alpha function

$$h_e(t) = \frac{H_e}{\tau_e} t \exp(-t/\tau_e) \tag{3}$$

with magnitude H_e and time constant τ_e. Inhibitory synapses are similarly defined but with kernels $h_i(t)$ and parameters H_i, τ_i.

Single Region

The activity of a single neocortical unit is then defined by the convolution equations

$$\begin{aligned} v_i &= \gamma_3 s(\tilde{v}_p) \otimes h_e \\ v_s &= [u + \gamma_1 s(\tilde{v}_p)] \otimes h_e \\ v_{pe} &= \gamma_2 s(\tilde{v}_s) \otimes h_e \\ v_{pi} &= \gamma_4 s(\tilde{v}_i) \otimes h_i \\ v_p &= v_{pe} - v_{pi} \end{aligned} \tag{4}$$

where v_{pe} and v_{pi} are potentials at excitatory and inhibitory synapses in the pyramidal cell population, $\tilde{v}$ denotes the potential after a delay δ_{ii} due to signalling delays among the different populations within a single brain region. Following [2] a first order Taylor series approximation is used to capture these delays, $\tilde{v} = v - \delta_i \dot{v}$. The connection strengths among neural populations are

specified by the parameters $\gamma_{1..4}$. These within-region values are also referred to as the 'intrinsic connectivity'.

Each of the above convolution equations can be written as a second order differential equation, or two first order DEs, as shown in [3] (see also next section). Thus a single cortical unit has $N_x = 9$ state variables. The input to the cortical region is a surrogate for event-related subcortical brain activity and is specified by a Gaussian function peaking at 64ms post-stimulus with width 16ms.

Differential equations

Each synapse

$$\begin{aligned} v_{out}(t) &= h_e(t) \otimes s(v_{in}(t)) && (5) \\ h_e(t) &= \frac{H_e}{\tau_e} t \exp(-t/\tau_e) && (6) \end{aligned}$$

can be implemented with a second order DE or two first order DEs [3]

$$\begin{aligned} \dot{v}_{out} &= c_{out} && (7) \\ \dot{c}_{out} &= \frac{H_e}{\tau_e} s(v_{in}) - \frac{2}{\tau_e} c_{out} - \frac{1}{\tau_e^2} v_{out} && (8) \end{aligned}$$

where c_{out} is the current flowing through the synapse. Hence each synapse gives rise to two DEs. The convolution equations that define neural masses then become a set of differential equations. For a single cortical unit we have

$$\begin{aligned} \dot{v}_s &= c_s && (9) \\ \dot{v}_{pe} &= c_{pe} \\ \dot{v}_{pi} &= c_{pi} \\ \dot{c}_s &= \frac{H_e}{\tau_e} \gamma_3 (u + \gamma_1 s(v_p)) - \frac{2}{\tau_e} c_s - \frac{1}{\tau_e^2} v_s \\ \dot{c}_{pe} &= \frac{H_e}{\tau_e} \gamma_2 s(v_s) - \frac{2}{\tau_e} c_{pe} - \frac{1}{\tau_e^2} v_{pe} \\ \dot{c}_{pi} &= \frac{H_i}{\tau_i} \gamma_4 s(v_i) - \frac{2}{\tau_i} c_{pi} - \frac{1}{\tau_i^2} v_{pi} \\ \dot{v}_i &= c_i \\ \dot{c}_i &= \frac{H_e}{\tau_e} \gamma_3 s(v_p) - \frac{2}{\tau_e} c_i - \frac{1}{\tau_e^2} v_i \\ \dot{v}_p &= c_{pe} - c_{pi} \end{aligned}$$

Two Regions

This two region model with forward connection a_{21} (from region 1 to 2) and backward connection a_{12} is shown in Fig. 1. For a two-region model these connectivities are stored in a 2-by-2 A matrix. For our 6 region models this is a 6-by-6 matrix.

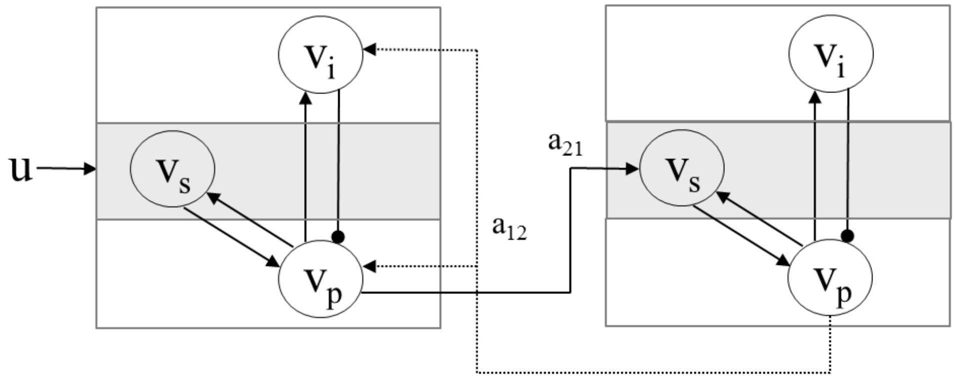

Fig. 1: Neural mass model of two cortical regions in a hierarchical network where v_s, v_i and v_p denote average membrane voltages in populations of stellate cells, inhibitory interneurons and pyramidal cells. The first unit receives tha-lamic input u, and projects output $v_p(1)$ via a forward connection of strength a_{21} to region 2. The second unit produces output $v_p(2)$ and projects it via a backward connection of strength a_{12} to region 1.

The equations for the first cortical region are

$$\begin{aligned} v_i(1) &= [a_{12}s(\bar{v}_p(2)) + \gamma_3 s(\tilde{v}_p(1))] \otimes h_e \\ v_s(1) &= [u + \gamma_1 s(\tilde{v}_p(1))] \otimes h_e \\ v_{pe}(1) &= [a_{12}s(\bar{v}_p(2)) + \gamma_2 s(\tilde{v}_s(1))] \otimes h_e \\ v_{pi}(1) &= \gamma_4 s(\tilde{v}_i(1)) \otimes h_i \\ v_p(1) &= v_{pe}(1) - v_{pi}(1) \end{aligned} \tag{10}$$

where $v_i(1)$, $v_s(1)$ and $v_p(1)$ are average membrane potentials of inhibitory interneuron, stellate cell and pyramidal cell populations (v_{pi} and v_{pe} are potentials at inhibitory and excitatory synapses within the pyramidal population). Here u is the known input variable, a surrogate for event-related subcortical brain activity and is specified by a Gaussian function peaking at 64ms post-stimulus with width 16 ms. Parameters γ_1 to γ_4 denote within-unit or 'intrinsic' connection strengths. The firing rate functions $s()$ and synaptic kernels h_e and h_i are defined in [2] and $\otimes$ denotes the convolution operator (these types of models are also known as convolution models [4]).

For the second region we have

$$\begin{aligned} v_i(2) &= \gamma_3 s(\tilde{v}_p(2)) \otimes h_e \\ v_s(2) &= [a_{21}s(\bar{v}_p(1)) + \gamma_1 s(\tilde{v}_p(2))] \otimes h_e \\ v_{pe}(2) &= \gamma_2 s(\tilde{v}_s(2)) \otimes h_e \\ v_{pi}(2) &= \gamma_4 s(\tilde{v}_i(2)) \otimes h_i \\ v_p(2) &= v_{pe}(2) - v_{pi}(2) \end{aligned} \tag{11}$$

In these equations $\tilde{v}$ denotes the potential after a delay τ_{ii} due to signalling delays among the different cell populations within a cortical region. A first order

Taylor series, $\tilde{v} = v - \tau_{ii}\dot{v}$ is used to capture these within-region (or 'intrinsic') delays (where $\dot{v}$ denotes the derivative with respect to time). Similarly, $\bar{v} = v - \tau_{ij}\dot{v}$ captures the 'extrinsic' delay, τ_{ij}, from region j to i.

References

[1] Jansen B, Rit V (1995) Electroencephalogram and visual evoked potential generation in a mathematical model of coupled cortical columns. *Biol Cybern* **73**, 357–366.

[2] David O, Kiebel S, Harrison L, Mattout J, Kilner J, Friston K (2006) Dynamic causal modeling of evoked responses in EEG and MEG. *Neuroimage* **30**, 1255–1272.

[3] Grimbert F, Faugeras O (2006) Bifurcation analysis of Jansen's neural mass model. *Neural Comput* **18**, 3052–3068.

[4] Moran R, Pinotsis D, Friston K (2013) Neural masses and fields in dynamic causal modeling. *Front Comput Neurosci* **7**, 57.
