# Supplementary material for: Dynamic Causal Modeling of Preclinical Autosomal-Dominant Alzheimer’s Disease
Source: J Alzheimers Dis. 2018 Sep 11;65(3):697–711. doi: 10.3233/JAD-170405 (PMC6923812; doi:10.3233/JAD-170405)
Supplement: Supplementary Material 2 [file jad-65-jad170405-s002.pdf]

# Supplementary Material 2

## Dynamic Causal Modeling of Preclinical Autosomal-Dominant Alzheimer's Disease

## Cognitive Trajectories

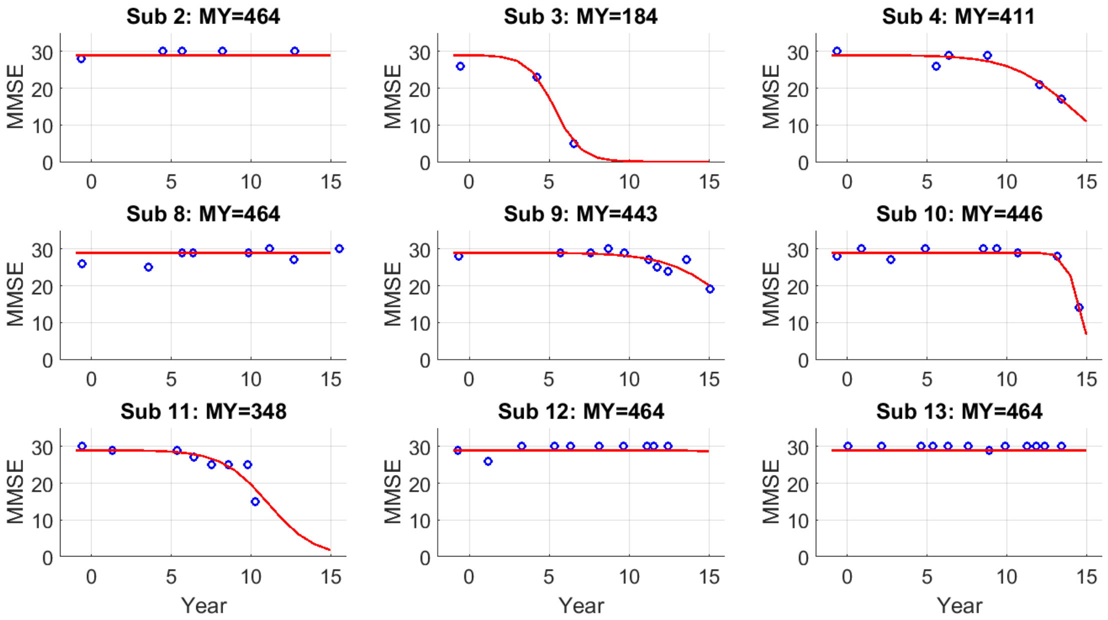


**Fig. 1. MMSE Trajectories.** Mini-Mental State Exam (MMSE) trajectories during follow-up period for 9 subjects from the PreC group (the ones not shown in the main paper). The x-axis labels Year with 0 corresponding to 2000. The EEG data were acquired in 1999. Blue dots denote measured MMSE scores and the red line indicates the trajectory estimated using a logistic decay model. The MY values above each plot correspond to MMSE-Years, computed as the integral under the curve.
