# Supplementary material for: Dynamic Causal Modeling of Preclinical Autosomal-Dominant Alzheimer’s Disease
Source: J Alzheimers Dis. 2018 Sep 11;65(3):697–711. doi: 10.3233/JAD-170405 (PMC6923812; doi:10.3233/JAD-170405)
Supplement: Supplementary Material 3 [file jad-65-jad170405-s003.pdf]

Supplementary Material 3

Dynamic Causal Modeling of
Preclinical Autosomal-Dominant Alzheimer's
Disease

Source Reconstruction

Multiple Sparse Priors

Following Bobes et al. [1] we used a concentric spheres approximation for the electromagnetic forward model. This comprised four concentric spheres as described in Litvak et al. [2]. We used the source reconstruction approach employed in Brown and Friston [3] and described in Litvak and Friston [4] which used Multiple Sparse Priors (MSP) and group constraints. This approach optimizes prior covariance constraints on sources and subjects and provides source estimates on a cortical mesh. We used the Greedy Search (GS) option to estimate the covariance components. We used a mesh comprising 8196 dipoles and a time period from -100 to 600 ms post stimulus onset for each subject.

Group Inference

Contrasts were then created of log power differences in the 50 to 150 ms window (i.e., collapsed over group and congruency) versus baseline (-100 to 0 ms) for each subject. These contrast images were then smoothed with a 12 mm Gaussian kernel to create a 3D volumetric NIFTI image for each subject. These were then entered into a one-sample t-test design in SPM and we tested for changes in EEG power with respect to baseline using an F-contrast (i.e., looking for two-sided effects; power increases or decreases with respect to baseline). We found activation of left and right middle occipital gyrus (MOG) e.g., $[-28, -86, 30]$ and $[22, -78, 22]$ significant at $p < 0.05$ Family Wise Error (FWE) peak and cluster level. The activated voxels are shown in Fig. 1.

References

[1] Bobes M, Garcia Y, Lopera F, Quiroz Y, Galan L, Vega M, Trujillo N, Valdes-Sosa M, Sosa PV (2010) ERP generator anomalies in presymptomatic carriers of the Alzheimer's disease E280A PS-1 mutation. *Hum Brain Mapp* **31**, 247–275.

[2] Litvak V, Mattout J, Kiebel S, Phillips C, Henson R, Kilner J, Barnes G, Oostenveld R, Daunizeau J, Flandin G, Penny W, Friston K (2011) EEG

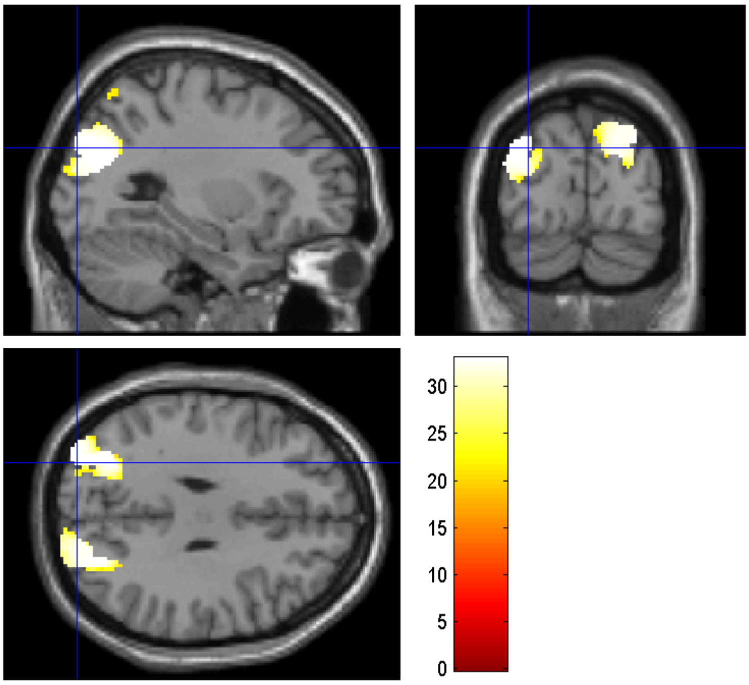

Fig. 1. **F-Statistic Map.** Values larger than $F = 20.5$ correspond to a FWE corrected height threshold of 0.05. The activation regions are left and right Middle Occipital Gyrus.

and MEG data analysis in SPM8. *Comput Intell Neurosci* **2011,** 852961.

[3] Brown H, Friston K (2012) Dynamic causal modelling of precision and synaptic gain in visual perception - an EEG study. *Neuroimage* **63**, 223–231.

[4] Litvak V, Friston K (2008) Electromagnetic source reconstruction for group studies. *Neuroimage* **42**, 1490–1498.
