# Supplementary material for: Dynamic Causal Modeling of Preclinical Autosomal-Dominant Alzheimer’s Disease
Source: J Alzheimers Dis. 2018 Sep 11;65(3):697–711. doi: 10.3233/JAD-170405 (PMC6923812; doi:10.3233/JAD-170405)
Supplement: Supplementary Material 4 [file jad-65-jad170405-s004.pdf]

Supplementary Material 4
Dynamic Causal Modeling of Preclinical Autosomal-Dominant Alzheimer's Disease

DCM Diagnostics

We now present some diagnostics for the M7 models on which we have reported effects of group, congruency and correlation with cognitive outcome.

Percent Variance Explained

A potential concern when comparing DCM parameter estimates is that spuri-ous differences may arise due to consistent differences in the nonlinear model estimation (instantiated using the VL algorithm). For example, if models don't fit well parameters will remain at their prior values, and if this only happens for one of the groups/conditions an artefactual difference could be reported. However, this was not the case with our data.

The average percent variance explained for the NonC and PreC groups was 84.6% and 84.8% respectively, and there was no significant difference ($p = 0.95$, $t = 0.06$, $df = 54$). Similarly, the average percent variance explained for the congruent and incongruent conditions was 83.9% and 85.3% and there was no significant difference ($p = 0.23$, $t = 1.24$, $df = 27$).

Source Locations

As described in the Methods section, DCM for ERP allows for the position of source locations to be optimized [1]. We now report inferences on how far the sources moved during optimization. This is computed as the Euclidean distance between the prior and posterior mean in mm. Table 1 reports average values separately for NonC and PreC groups and uses two-sample t-tests to test for differences. This indicates no significant differences between groups for any sources.

Looking at the values in Table 1, however, one can see that the MOG sources have moved less than the others. Statistical tests find this to be a significant effect. For example, Left MOG moved significantly less than Left IT, Right IT, Left MTL and Right MTL (p = 0.001, 0.007, 0.002, 0.05) but not Right MOG ($p = 0.43$). Similarly, Right MOG moved significantly less than Left IT, Right IT, Left MTL and Right MTL (p = 0.002, 0.001, 0.0001, 0.02) but not Left MOG ($p = 0.43$). Interestingly, the prior locations of IT and MTL were taken from a previous study [2] whereas MOG regions were identified using Statistical

Parametric Maps (SPMs) based on the current data and source space definition (see Supplementary Material 3 Text: Source Reconstruction).

Table 1: **Effect of Group of Source Locations.**

**Region**	**Group**		**Statistics**	
	NonC	PreC	p-value	t-value
Left MOG	1.6	2.6	0.17	-1.4
Right MOG	1.7	2.0	0.57	-0.6
Left IT	4.0	4.1	0.86	-0.18
Right IT	4.2	5.5	0.44	-0.78
Left MTL	3.9	3.7	0.90	0.12
Right MTL	4.6	2.5	0.12	1.6

The table reports the average Euclidean distance between prior and posterior mean source locations (in units of mm), and the significance thereof.

References

[1] Kiebel SJ, David O, Friston KJ (2006) Dynamic causal modelling of evoked responses in eeg/meg with lead field parameterization. *Neuroimage* **30**, 1273–1284.

[2] Bobes M, Garcia Y, Lopera F, Quiroz Y, Galan L, Vega M, Trujillo N, Valdes-Sosa M, Sosa PV (2010) ERP generator anomalies in presymptomatic carriers of the Alzheimer's disease E280A PS-1 mutation. *Hum Brain Mapp* **31**, 247–275.
