# Supplementary material for: Dynamic Causal Modeling of Preclinical Autosomal-Dominant Alzheimer’s Disease
Source: J Alzheimers Dis. 2018 Sep 11;65(3):697–711. doi: 10.3233/JAD-170405 (PMC6923812; doi:10.3233/JAD-170405)
Supplement: Supplementary Material 5 [file jad-65-jad170405-s005.pdf]

Supplementary Material 5
Dynamic Causal Modeling of Preclinical Autosomal-Dominant Alzheimer's Disease

Parameter Inferences

The first sections of the supplement test for effects of group, congruency and correlation with MMSE-Years using summary statistics. That is, entering posterior mean parameter estimates into group level t-tests. The last two sections describe tests using PEB.

Predicting Subsequent Cognitive Screening Measures

We regress the normalized MMSE-Years values onto the posterior mean value for each connection in the optimal model. These posterior means are those that reflect the positivity constraint in the connectivity values (similar results were found without exponentiation). More specifically, DCM defines latent variables $z^A_{ij} = \log A_{ij}$, $z^B_{ij} = \log B_{ij}$ and optimizes the z variables. These are then exponentiated to recover the A_{ij} and B_{ij} values. Table 1 shows all connections with $p < 0.05$ for all three conditions (congruent, incongruent, and both). Three of these correlations remain significant after correcting for the multiple comparisons over the 16 connections using Bonferroni. The PEB results presented in the main text are for the A matrix parameters (that is for the congruent condition).

Multiple Regression

MMSE-Years and age are negatively correlated ($r = -0.73$, $p = 0.005$) meaning that older subjects have smaller values (in the 16 year time horizon post EEG collection). MMSE-Years and task performance (as measured using d') are positively correlated ($r = 0.72$, $p = 0.006$) meaning that subjects who were better on the task have larger values. Age and task performance were negatively correlated ($r = -0.69$, $p = 0.01$) meaning that younger subjects performed better.

Given these correlations between MMSE-Years and age/performance we enter the age and performance variables into a multiple regression model which regresses MY onto connection value, age and performance. This allows us to test for correlations between MY and connectivity after controlling for age and performance effects. For the Left MTL to Right IT connection (for congruent items) we have a significant (partial) correlation ($r = -0.87$, $p = 0.0007$) and

Table 1: **Correlation with MMSE-Years.**

Pathway		Condition	Statistics	
From	To		R^2	p-value
LMTL	RIT	Both	0.51	0.006
		Congruent	0.63	0.001*
		Incongruent	0.38	0.02
RIT	RMTL	Both	0.60	0.001*
		Congruent	0.48	0.009
		Incongruent	0.60	0.002*
LMOG	RIT	Both	0.32	0.04
		Congruent	0.31	0.05
		Incongruent	0.31	0.05

Correlation between MMSE-Years, MY, and mean connection value over congruent and incongruent items (Both) or connection value over congruent items (Congruent) or incongruent (Incongruent) items. The asterisk denotes p-values that remain significant after correcting for the multiple comparisons over the 16 connections using Bonferroni.

for the Right IT to Right MTL connection (for congruent items) we also have a significant effect ($r = -0.72, p = 0.009$).

Group Differences

Table 2 shows results for the main effect of group (collapsing across congruency). Strikingly, all of these connections are larger in the PreC than NonC group. The posterior means used were those that reflected the positivity constraint (similar results were found without exponentiation).

Table 2: **Effect of Group.**

Pathway		Condition	Group Means, $\bar{a}$		Statistics
From	To		NonC	PreC	p-value
LMTL	RIT	Both	0.89	1.10	0.002*
		Congruent	0.91	1.09	0.01
		Incongruent	0.88	1.11	0.05
RIT	RMTL	Both	0.95	1.04	0.20
		Congruent	0.95	1.12	0.08
		Incongruent	0.94	0.96	0.89
LMOG	RIT	Both	0.89	1.01	0.02
		Congruent	0.93	1.01	0.15
		Incongruent	0.86	1.02	0.08

Two-sample t-tests.

Effect of congruency

The effect of congruency is characterised by values in the estimated modulatary connectivity matrix B, as described above. We use a liberal p-value threshold of 0.1. We use $\bar{b}$ to denote the average value of the modulatory connection, with values less (greater) than 1 indicating that the pathway is less (more) activated for incongruent versus congruent items. Here, the one sample t-tests were carried out on the z_{ij}^B values (as all estimates would be larger than zero after exponentiation and would therefore violate assumptions of the test).

Table 3: **Effect of Congruency.**

**Pathway**		**Group**	**Mean,** $\bar{b}$	**Statistics**
From	To			p-value
LMOG	LIT	Both	0.93	0.10
		NonC	0.92	0.09
		PreC	0.95	0.50
RMOG	LIT	Both	0.92	0.01
		NonC	0.89	0.02
		PreC	0.96	0.31
RIT	LMTL	Both*	0.93	0.12
		NonC	0.85	0.05
		PreC	1.02	0.50
RIT	RMTL	Both*	0.93	0.03
		NonC	0.97	0.62
		PreC	0.85	0.01

One-sample t-tests. Asterisks denote pathways with a significant group by congruency interaction (see text).

Strikingly, all of the $\bar{b}$ values are less than unity (except for one) indicating lower activation of pathways for incongruent versus congruent items. The only $\bar{b}$ value that is larger than 1 is the RIT to LMTL pathway in the PreC group. This was estimate to be even larger using PEB (see main text).

For the group by congruency interaction effect we have Right IT to Left MTL ($p = 0.04$). This pathway increased (wrt congruent) for PreC but decreased for NonC. We also have Right IT to Right MTL ($p = 0.09$) for which we have a large decrease for PreC and a small one for NonC.

The results of these statistical tests are largely in accord with the PEB-based methods presented in the main text. Minor differences are due to the following factors; (i) PEB iteratively udpates posterior mean parameter estimates using the posterior means (and covariances) of all subjects and a second level (group) model. The estimates of effects in the A and B matrices will therefore be different. (ii) PEB uses a different method of statistical testing. Either based on posterior probabilities of second level parameters to assess effects of group, congruence or group by congruency, or estimates of the Pearson correlation coefficient from leave-one-out (LOO) correlation. Note that these LOO-based correlation estimates produce slightly lower R^2 values. This is because they have been computed from "out-of-sample" data.

Effect of Congruency - assessed using PEB

The effects of congruency as assessed using PEB are reported in Table 4.

Table 4: **B matrix connections showing overall effect**

Pathway		Means	Statistics
From	To	b	P_{post}
RMOG	LIT	0.91	0.95
RIT	RMTL	0.89	0.97

Connections in this table have a posterior probability, P_{post}, greater than 0.95 of showing a modulatory effect (i.e., of congruency).

Effect of Age - assessed using PEB

We specifically tested for the effect of age on connectivity. First we constrained our search to the four connections that showed an effect of group. We found no correlations between multivariate predictions of age and empirical age in the PreC ($r = -0.07, p = 0.59$) or NonC ($r = -0.44, p = 0.95$) groups. Note the correlations here are between predictions of the left out sample value and actual sample value, so that a negative correlation implies an inconsistency and therefore lack of a significant effect. No significant correlations were found in either group when using pathways (iii - Left MTL to Right IT) and (iv - Right IT to Right MTL) individually or together.

We then looked more widely at all A and B parameters of the DCMs. For the NonC group we found a single (second-level regression) parameter that was significantly non-zero. This regression coefficient related the B parameter for the connection from Right IT to Left MTL to Age with older subjects having smaller B values (the posterior probability of this effect being non-zero is $p = 0.98$). Thus, the mismatch (or "incongruency") effect of Right IT on Left MTL is smaller in older subjects. This is reminiscent of DCM for Event-Related Field modeling of MEG data from an auditory mismatch negativity task in which older subjects had smaller feedforward connections that signaled a mismatch (in that case right Heschl's Gyrus to right Superior Temporal Gyrus connections)[1].

For the PreC group we found 4 connections showing a correlation with age. The first two show a negative correlation: (i) the B matrix value from Left MOG to Left IT ($p = 0.96$), (ii) the B matrix value from Left MOG to Right IT ($p = 0.95$). Thus, the mismatch effect on these connections is smaller in older subjects. The second two show a positive correlation: (iii) the A matrix value from Left IT to MTL ($p = 0.99$) and (iv) the A matrix value from Right IT to Right MTL ($p = 0.98$). Thus, these connections are larger in older subjects.

The above effects of age are based on the posterior probability of group level regression coefficients. We then used the more stringent test of association based on the leave-one-out PEB procedure [2]. None of the above effects survive this more stringent test.

References

[1] Moran R, Symmonds M, Dolan R, Friston K (2014) The brain ages optimally to model its environment: evidence from sensory learning over the adult lifespan. *PLoS ComputBiol* **10**, e1003422.

[2] Friston K, Litvak V, Oswal A, Razi A, Stephan K, van Wijk B, Ziegler G, Zeidman P (2016) Bayesian model reduction and empirical Bayes for group (DCM) studies. *Neuroimage* **128**, 413–431.
